# Horizontal and Vertical Defect Management with a Novel Degradable Pure Magnesium Guided Bone Regeneration (GBR) Membrane—A Clinical Case

**DOI:** 10.3390/medicina59112009

**Published:** 2023-11-15

**Authors:** Massimo Frosecchi

**Affiliations:** Department of Surgical and Diagnostic Sciences (DISC), University of Genoa, 16132 Genoa, Italy; massimofrosecchi@gmail.com

**Keywords:** periodontal diseases, bone regeneration, magnesium

## Abstract

*Background and objectives:* In guided bone regeneration (GBR), large defects comprising both horizontal and vertical components usually require additional mechanical support to stabilize the augmentation and preserve the bone volume. This additional support is usually attained by using non-resorbable materials. A recently developed magnesium membrane presents the possibility of providing mechanical support whilst being completely resorbable. The aim of this case report was to describe the application and outcome of the magnesium membrane in combination with a collagen pericardium membrane for GBR. *Materials and methods:* A 74 year old, in an otherwise good general health condition, was presented with stage 2 grade A periodontitis and an impacted canine. After extraction of the impacted canine, a defect was created with both vertical and horizontal components. The defect was augmented using the magnesium membrane to create a supportive arch to the underlying bone graft and a collagen pericardium membrane was placed on top to aid with the soft tissue closure. *Results:* Upon reentry at 8 months, complete resorption of the magnesium devices was confirmed as there were no visible remnants remaining. A successful augmentation outcome had been achieved as the magnesium membrane in combination with the collagen membrane had maintained the augmented bone well. Two dental implants could be successfully placed in the healed augmentation. *Conclusions:* In this case, the magnesium membrane in combination with a collagen pericardium membrane presented a potentially viable alternative treatment to titanium meshes or titanium-reinforced membranes for the augmentation of a defect with both horizontal and vertical components that is completely resorbable. It was demonstrated that it is possible to attain a good quality and quantity of bone using a resorbable system that has been completely resorbed by the time of reentry.

## 1. Introduction

Reconstructive treatments, such as guided bone regeneration (GBR), have become an integral part of implant/prosthetic rehabilitation processes over the years [1]. The principle of GBR relies on the use of a bone augmentation material to provide a scaffold for the new bone to infiltrate [2], and an overlying barrier membrane to seclude the defect space from the fast-growing epithelial cells of the overlying gingival tissue [3].

GBR has been applied to correcting bone defects with horizontal, and more recently, vertical components [4,5,6,7]. Depending on the characteristics of the defect, the choices of the materials used for the bone graft and the membrane are very important for a successful outcome. For instance, collagen membranes are often used as they provide excellent biocompatible properties and are resorbed into the soft tissue whilst maintaining a barrier function [8]. However, a low mechanical strength means that they are unsuitable for large and vertical defects, as there is the risk that they might collapse into the defect space [9,10].

For large-sized defects with either vertical or combined horizontal and vertical components, the defect space requires additional mechanical support to enable successful bony regeneration. In these situations, mechanical support has previously been attained via the use of screws, titanium-reinforced non-resorbable membranes, or customized titanium grids and plates [6,11,12,13,14,15]. These materials have the advantage that they can provide a continuous support to the defect site for as long as is required, and can remain in situ for longer should the healing situation require it. However, as these materials are non-resorbable, it is necessary to remove them by means of an uncovering surgery. This can potentially require a larger flap upon reentry to recover all of the non-resorbable material, thereby increasing patient morbidity.

Recently, a mechanically stable but completely resorbable magnesium metal membrane has been developed that could provide an alternative solution for the treatment of these defects [14,16]. Magnesium is a resorbable biomaterial that is already used in medical devices for orthopedic and cardiovascular applications [17,18]; however, the magnesium membrane was the first magnesium medical device to receive a CE mark in 2021 [19]. Magnesium is a biometal that degrades when placed in the body into non-toxic biocompatible byproducts that are then resorbed [20]. As it degrades, it releases magnesium ions, which are naturally prevalent within the body and are present in almost every cell [21]. Its initial mechanical strength provides a rigid three-dimensional volumetric maintenance to stabilize the underlying bone graft during the critical healing period, yet reabsorbs in the subsequent months following implantation in a complete and predictable way [14,16,22,23,24,25,26,27]. The membrane has undergone substantial preclinical research [14,16], and since its CE approval, its clinical performance has been reported in several case studies [24,25,26,27].

The aim of this clinical case was to investigate the application of the magnesium membrane to support a collagen pericardium membrane for the treatment of a combined vertical and horizontal bony defect and its ability to maintain bone volume.

## 2. Case Presentation

The patient, aged 74, was a non-smoker in otherwise good general health, but had stage 2 grade A periodontitis, that was under maintenance treatment [28]. The patient came to our observation to rehabilitate the upper left posterior sector. In this area, there was mobility of a prosthetic bridge supported by natural pillars 27 and 24, with 23, 25, and 26 as pontic components. Clinical examination demonstrated that mobility was mainly caused by tooth 24, treated endodontically with a periodontal probing depth greater than 9 mm. Tooth 27 also had a periodontal pocket of 5 mm.

**Figure 1:**
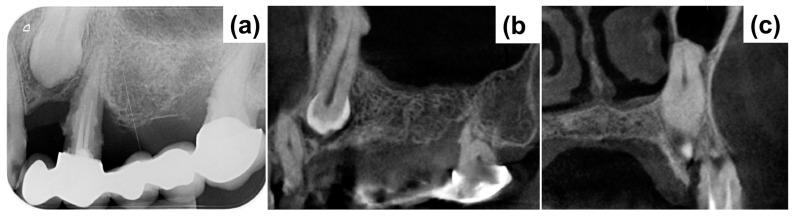
(**a**) Initial X-ray demonstrating peripheral bone loss to the first premolar 24. (**b**,**c**) cone beam computed tomography (CBCT) demonstrating severe bone loss around 24 and the positioning of the impacted canine 23.

Periapical X-ray (Figure 1a) showed periapical bone loss to the first premolar (24), which appears to have been treated endodontically in an incongruous way. The second molar (27) showed a mesial bone defect. The X-ray also showed the presence of an impacted canine (23) with increased peri-coronal space, indicating a possible bacterial contamination. In order to evaluate the impacted canine and set the treatment plan, cone beam computed tomography (CBCT) was executed (Figure 1b,c). The CBCT examination highlighted an area of radiotransparency for the impacted canine and the complete loss of bone support for tooth 24. It also highlighted an alteration in the shape of the apex of the canine in contact with the maxillary sinus.

Considering the clinical and the instrumental examinations, extraction of teeth 24 and 23 was indicated, while 27 was considered treatable from the periodontal point of view. It was therefore necessary to replace teeth 23, 24, 25, and 26.

Molar 27 was treated with scaling and root planing and the site was reevaluated after 2 months. Upon reevaluation, there was no bleeding on probing and the probing depth reduced from 5 mm to 3 mm with a furcation probing grade of 1.

**Figure 2:**
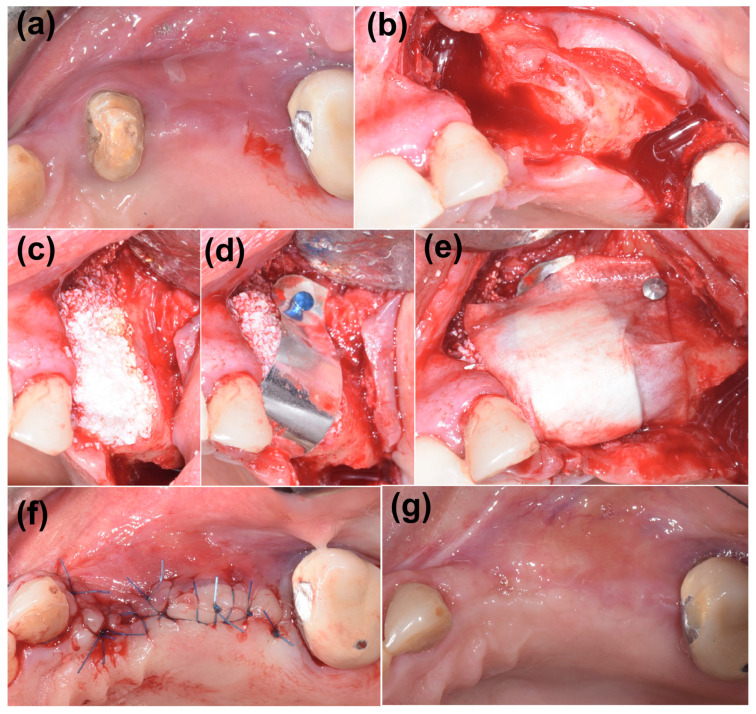
Augmentation of the bony defect. (**a**) Initial clinical situation, (**b**) bone defect after the extraction on 23 and 24, presenting a vertical and horizontal component, (**c**) application of the bovine bone graft, (**d**) placement of the magnesium membrane, cut into a rounded strip and bent over the defect to provide a supporting arch, (**e**) positioning of a pericardium membrane, (**f**) first intention closure, (**g**) healed site at 8 months.

The planned extraction of the impacted teeth 23 and 24 would leave a complex and deep bone defect, with horizontal and vertical components. Therefore, the first surgical step was dedicated to the removal of 23 and 24 and subsequent defect management. The resulting bone defect was considered complex given the deep vestibular bone loss at location 23 and the vertical component of the defect between areas 23 and 24 (Figure 2b).

Heterologous bone (cerabone^®^, botiss biomaterials GmbH, Zossen, Germany) in a granular form was used as the bone substitute to augment the defect (Figure 2c). The sintered bovine bone provides excellent volume stability with only superficial degradation [29]. It also has a reported excellent hydrophilicity [30], which promotes the infiltration of blood and precursor cells for promoting vascularization [31,32] and osseous integration [33].

Due to the presence of a vertical component, a rigid magnesium membrane (NOVAMag^®^ membrane, botiss biomaterials GmbH, Zossen, Germany) was necessary to obtain a stable volume, able to resist the pressure of the overlying soft tissue when closing the flap. This membrane, despite having a typically metallic appearance and rigidity, has the ability to be completely reabsorbed in approximately 4 months. This device was bent, cut, and refined, and then adapted to create an arch over the defect (Figure 2d). The membrane was secured to both the buccal and palatal walls using titanium pins (Titan-Pins, Ustomed, Tuttlingen, Germany). The magnesium membrane was covered with a resorbable membrane made of porcine pericardium (Jason^®^, botiss biomaterials GmbH, Zossen, Germany) in order to allow an easier adaptation of the overlying flap (Figure 2e). The collagen membrane was also stabilized using the metal pins. The first intention closure completed the first surgical phase (Figure 2f). No removable temporary was applied so as not to interfere with the healing phase.

**Figure 3:**
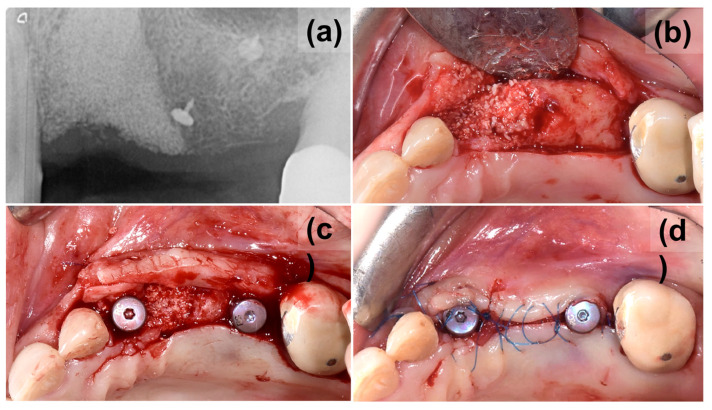
Reentry at 8 months, (**a**) control periapical X-ray, presenting good maintenance of bone volume, (**b**) flap elevation for implant placement, (**c**) implant placement, (**d**) non-submerged healing with the application of healing screws.

During the follow-up period, there were no complications and the soft tissue healed well, leaving a thick keratinized tissue above the treated site (Figure 2g). A periapical X-ray was taken after 8 months to assess the stability of the grafted material and bone (Figure 3a). The X-ray confirmed good maintenance of the augmented bone volume. Upon reentry, a muco-periosteal flap was elevated and successful bone regeneration was proven (Figure 3b). Two implants were placed at the sites 23 and 26 (BLT, Straumann, Switzerland), and healing abutments were placed immediately for non-submerged healing (Figure 3c,d).

**Figure 4:**
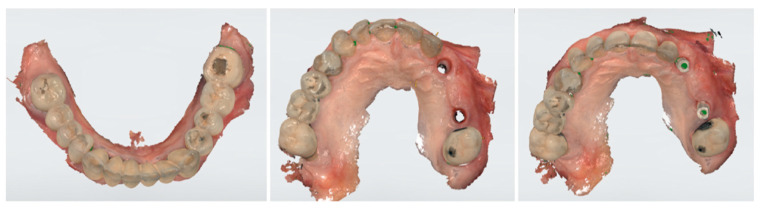
Optical impressions for completion of the final prosthesis.

**Figure 5:**
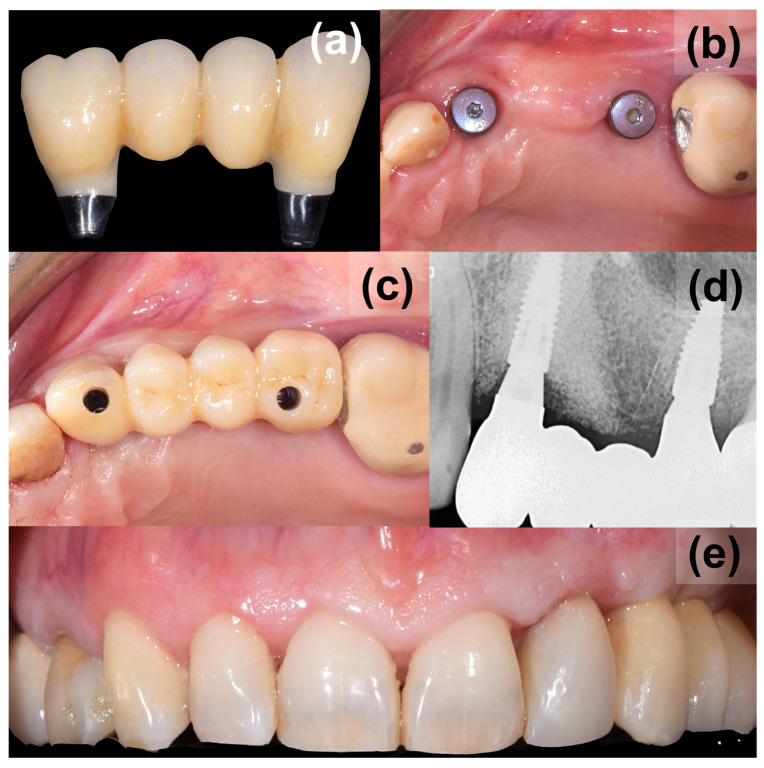
Application of final prosthesis, (**a**) a screw retained three-unit bridge made of monolithic zirconia, (**b**) final appearance of augmented site, (**c**) applied bridge, (**d**) control X-ray, (**e**) final aesthetics from the buccal view.

After another 3 months, following the verification of osseointegration, optical impressions were taken for the completion of the final prosthesis (Figure 4). After normal prosthetic phases, a screw retaining a three-unit bridge made of monolithic zirconia was applied (Figure 5). The occlusion and soft tissue compression were verified. After delivery, the occlusal function and soft tissue stability were monitored in the following months.

## 3. Discussion

Regenerative treatments associated with implant treatments have now become extremely common. They are rather standardized treatments and are based on the creation or maintenance of volume using autological, homologous, heterologous, or synthetic type bone augmentation materials, as well as resorbable or non-reabsorbing barrier membranes. Complex defects, especially those that comprise both horizontal and vertical components, require rigid devices to provide volumetric maintenance [34]. For this purpose, customized or standard titanium barriers, titanium-reinforced membranes, or screws are often used [35]. However, the main disadvantage of these techniques is that they rely on non-resorbable materials to provide the required structural support. Although the rigid properties provide sustained mechanical support, they can also cause mechanical irritation to the mucosal flap, leading to exposure [36]. Additionally, dense fibrous tissue can form under titanium meshes that also integrates with the mesh, making them difficult to extract [37].

Resorbable barrier membranes made from collagen are advantageous in GBR as they are highly biocompatible and are replaced with soft tissues as they are resorbed by the body [8]. Therefore, there is no need for their extraction upon reentry. However, due to their poor mechanical strength in comparison to titanium, they risk collapse into the defect void and are unsuitable for large defects with vertical components [9,10].

Despite the problems associated with titanium meshes and titanium-reinforced membranes, for large augmentations, they are an appealing option as they provide continuous mechanical support to the defect until they are surgically removed after the healing period. When compared to collagen membranes for vertical augmentations, Konstantinidis et al. [38] reported significantly more bone gain in the titanium mesh group compared to the collagen group. It has also been reported that there is no significant difference in bone gain between titanium-reinforced PTFE membranes and titanium meshes [39]. Due to the high rate of wound dehiscence associated with titanium meshes [40], some studies have investigated the application of collagen membranes over titanium meshes, with varying results [37,40,41].

Hence, a fully resorbable system that is mechanically strong presents an idealistic option. This would limit patient morbidity during reentry as the devices do not need to be extracted after the healing period, thereby reducing surgical times, whilst also providing the required mechanical support during the critical healing period for optimal bony ingrowth into the defect space. The recently developed magnesium membrane combines the properties of mechanical stability and resorption [14], which makes it a potential alternative to the other established treatment techniques. This clinical case investigated the potential for a magnesium membrane in combination with a collagen membrane to treat complex defects with both horizontal and vertical components.

The purpose of reinforcing the collagen membrane was to benefit from the known biocompatibility of the collagen, as well as the mechanical support of the magnesium metal. Additionally, the application of the collagen membrane over the magnesium membrane was intended to counteract the previously reported problems associated with the rigidity of the titanium meshes, which could potentially cause soft tissue complications.

A previous in vivo study demonstrated the potential of the magnesium membrane for GBR treatments in beagle dogs. In the reported study, the membrane maintained a barrier function in a GBR model as efficiently as a collagen membrane control group [16]. The potential for the membrane has also been demonstrated clinically [24,26,27].

In the selected case, after extraction of teeth 23 and 24, a complex vertical defect was created. The management of cases with bone defects resulting from complex extractions must always be carefully evaluated, not only in terms of the techniques and materials to be applied but also in terms of timing. Another factor to consider in these cases is the number of surgical steps required. This can vary based on many factors. In a non-esthetic region, it is sometimes possible to limit them, provided that they do not undermine the biological principles underlying the implant treatments.

**Figure 6:**
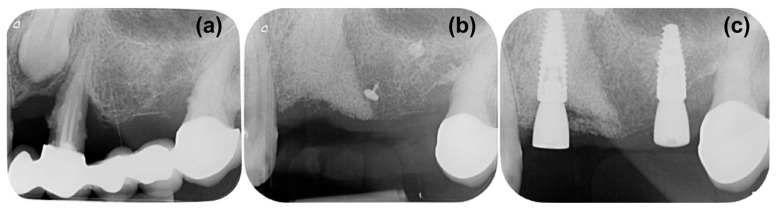
Comparison of periapical X-rays during different phases of the treatment, (**a**) the initial situation, (**b**) control X-ray at 8 months, prior to reentry, (**c**) post operative X-ray after implant placement.

It was determined that additional support would be necessary to maintain the bone volume in the augmented defect. Using a magnesium membrane, a resorbable supporting arch was built over the augmented bone, stabilizing the graft. The mechanical stability of the membrane was intended to resist the compressive forces of the overlying soft tissue to provide the maximum volume for new bone to grow into and occupy [42]. The mechanical support that can be provided by the magnesium membrane has previously been demonstrated by Elad et al. [26], who used the membrane to bridge the buccal or palatal walls in compromised sockets. Using the membrane in this method supported the graft and therefore preserved the ridge height during the healing period. It was also reported that there was a formation of a cortical plate in the position of the augmented bone. The mechanical stability of the magnesium membrane was also demonstrated in the presented clinical case, as it provided a stable structure that enabled new bone to grow both horizontally and vertically into the defect space (Figure 6a,b). The bone volume was preserved during the extended healing period, after which the placement of two dental implants into the augmented bone was possible.

To aid with the closing of the soft tissue, a collagen pericardium membrane was placed over the magnesium membrane arch. During the follow-up period, there were no complications with the soft tissue that are commonly associated with rigid membranes, such as wound dehiscence [35]. Instead, the soft tissue healed well, presenting a thick keratinized soft tissue prior to reentry (Figure 2g).

Palkovics et al. [27] also previously reported the successful combined use of the magnesium membrane with a heterologous collagen soft tissue graft, although in their reported case, the membranes were applied using the tunnel technique. Using this technique, the magnesium membrane was positioned under the periosteum with the collagen soft tissue graft over the top to improve the soft tissue contour. In their reported case, there were also no soft tissue complications, and after 6 months, 0.93 mm, 1.23 mm, and 1.38 mm horizontal bone gain was achieved 1, 2, and 3 mm apically to the alveolar crest.

During the degradation of magnesium metal, hydrogen gas is released, which is then absorbed by the body [43,44]. With the release of hydrogen gas from the membrane, there is the potential that a build-up of gas could lead to the separation of the wound along the line of the suture, causing a wound dehiscence. Wound dehiscence was previously reported in an in vivo study using the membrane [16]. However, in this instance, and in concurrence with the published cases [24,25,26,27], there were no clinical observations associated with the release of hydrogen gas.

## 4. Conclusions

Overall, the presented case demonstrates the potential for a magnesium membrane to reinforce collagen membranes for complex defects with both horizontal and vertical components. The chosen combination of materials preserved the bone volume, enabling stable implant placement upon reentry. Additionally, in this clinical case, a wound dehiscence did not occur, which is a clinical complication that is commonly associated with using rigid materials. Therefore, there is the potential that the application of a collagen membrane over the rigid magnesium prevented this from happening. Further studies are required to determine the full potential of this technique in providing an alternative to titanium meshes or titanium-reinforced membranes, such as limitations in defect sizes, as well as its ability to prevent soft tissue complications.

The advantages of the selected treatment based on this clinical case were as follows:

1. Execution of only two surgical phases, limiting discomfort and invasiveness.
2. Use of materials that are completely resorbable and are capable of handling complex bone defects with a vertical component.

## Data Availability

All data that were used for this study are included in the paper.

## References

[1] Khojasteh A., Kheiri L., Motamedian S., Khoshkam V. (2017). Guided bone regeneration for the reconstruction of alveolar bone defects. Ann. Maxillofac. Surg..

[2] Perić Kačarević Ž., Rider P., Alkildani S., Retnasingh S., Pejakić M., Schnettler R., Gosau M., Smeets R., Jung O., Barbeck M. (2020). An introduction to bone tissue engineering. Int. J. Artif. Organs.

[3] Scantlebury T., Ambruster J. (2012). The development of guided regeneration: Making the impossible possible and the unpredictable predictable. J. Evid. Based Dent. Pract..

[4] Elnayef B., Monje A., Albiol G., Galindo-Moreno P., Wang H.-L., Hernández-Alfaro F. (2017). Vertical Ridge Augmentation in the Atrophic Mandible: A Systematic Review and Meta-Analysis. Int. J. Oral Maxillofac. Implant..

[5] Plonka A., Urban I., Wang H.-L. (2018). Decision Tree for Vertical Ridge Augmentation. Int. J. Periodontics Restor. Dent..

[6] Ricci L., Perrotti V., Ravera L., Scarano A., Piattelli A., Iezzi G. (2013). Rehabilitation of Deficient Alveolar Ridges Using Titanium Grids before and Simultaneously with Implant Placement: A Systematic Review. J. Periodontol..

[7] Urban I.A., Montero E., Monje A., Sanz-Sánchez I. (2019). Effectiveness of vertical ridge augmentation interventions: A systematic review and meta-analysis. J. Clin. Periodontol..

[8] Bunyaratavej P., Wang H.-L. (2001). Collagen Membranes: A Review. J. Periodontol..

[9] Naenni N., Sapata V., Bienz S.P., Leventis M., Jung R.E., Hämmerle C.H.F., Thoma D.S. (2018). Effect of flapless ridge preservation with two different alloplastic materials in sockets with buccal dehiscence defects—Volumetric and linear changes. Clin. Oral Investig..

[10] Mir-Mari J., Wui H., Jung R.E., Hämmerle C.H.F.F., Benic G.I., Wui H., Jung R.E., Hämmerle C.H.F.F., Benic G.I. (2016). Influence of blinded wound closure on the volume stability of different GBR materials: An in vitro cone-beam computed tomographic examination. Clin. Oral Implant. Res..

[11] Hameed M.H., Gul M., Ghafoor R., Khan F.R. (2019). Vertical Ridge Gain with Various Bone Augmentation Techniques: A Systematic Review and Meta-Analysis. J. Prosthodont..

[12] Jung R.E., Brügger L.V., Bienz S.P., Hüsler J., Hämmerle C.H.F., Zitzmann N.U. (2021). Clinical and radiographical performance of implants placed with simultaneous guided bone regeneration using resorbable and nonresorbable membranes after 22–24 years, a prospective, controlled clinical trial. Clin. Oral Implant. Res..

[13] Zhang M., Zhou Z., Yun J., Liu R., Li J., Chen Y., Cai H., Jiang H.B., Lee E.-S., Han J. (2022). Effect of Different Membranes on Vertical Bone Regeneration: A Systematic Review and Network Meta-Analysis. Biomed. Res. Int..

[14] Rider P., Kačarević Ž.P., Elad A., Tadic D., Rothamel D., Sauer G., Bornert F., Windisch P., Hangyási D.B., Molnar B. (2022). Biodegradable magnesium barrier membrane used for guided bone regeneration in dental surgery. Bioact. Mater..

[15] Rossi R., Ghezzi C., Tomecek M. (2020). Cortical lamina: A new device for the treatment of moderate and severe tridimensional bone and soft tissue defects. Int. J. Esthet. Dent..

[16] Rider P., Kačarević Ž.P., Elad A., Rothamel D., Sauer G., Bornert F., Windisch P., Hangyási D., Molnar B., Hesse B. (2022). Analysis of a Pure Magnesium Membrane Degradation Process and Its Functionality When Used in a Guided Bone Regeneration Model in Beagle Dogs. Materials.

[17] May H., Alper Kati Y., Gumussuyu G., Yunus Emre T., Unal M., Kose O. (2020). Bioabsorbable magnesium screw versus conventional titanium screw fixation for medial malleolar fractures. J. Orthop. Traumatol..

[18] Cerrato E., Barbero U., Gil Romero J.A., Quadri G., Mejia-Renteria H., Tomassini F., Ferrari F., Varbella F., Gonzalo N., Escaned J. (2019). Magmaris^™^ resorbable magnesium scaffold: State-of-art review. Future Cardiol..

[19] Zan R., Shen S., Huang Y., Yu H., Liu Y., Yang S., Zheng B., Gong Z., Wang W., Zhang X. (2023). Research hotspots and trends of biodegradable magnesium and its alloys. Smart Mater. Med..

[20] Zheng Y.F., Gu X.N., Witte F. (2014). Biodegradable metals. Mater. Sci. Eng. R Rep..

[21] Gröber U., Schmidt J., Kisters K. (2015). Magnesium in prevention and therapy. Nutrients.

[22] Kačarević Ž.P., Rider P., Elad A., Tadic D., Rothamel D., Sauer G., Bornert F., Windisch P., Hangyási D.B., Molnar B. (2022). Biodegradable magnesium fixation screw for barrier membranes used in guided bone regeneration. Bioact. Mater..

[23] Yan Z.Y., Zhu J.H., Liu G.Q., Liu Z.C., Guo C.B., Cui N.H., Han J.M. (2022). Feasibility and Efficacy of a Degradable Magnesium-Alloy GBR Membrane for Bone Augmentation in a Distal Bone-Defect Model in Beagle Dogs. Bioinorg. Chem. Appl..

[24] Blašković M., Butorac Prpić I., Blašković D., Rider P., Tomas M., Čandrlić S., Botond Hangyasi D., Čandrlić M., Perić Kačarević Ž. (2023). Guided Bone Regeneration Using a Novel Magnesium Membrane: A Literature Review and a Report of Two Cases in Humans. J. Funct. Biomater..

[25] Blašković M., Blašković D., Hangyasi D.B., Peloza O.C., Tomas M., Čandrlić M., Rider P., Mang B., Kačarević Ž.P., Trajkovski B. (2023). Evaluation between Biodegradable Magnesium Metal GBR Membrane and Bovine Graft with or without Hyaluronate. Membranes.

[26] Elad A., Rider P., Rogge S., Witte F., Tadić D., Kačarević Ž.P., Steigmann L. (2023). Application of Biodegradable Magnesium Membrane Shield Technique for Immediate Dentoalveolar Bone Regeneration. Biomedicines.

[27] Palkovics D., Rider P., Rogge S., Kačarević Ž.P., Windisch P. (2023). Possible Applications for a Biodegradable Magnesium Membrane in Alveolar Ridge Augmentation—Retrospective Case Report with Two Years of Follow-Up. Medicina.

[28] Tonetti M.S., Greenwell H., Kornman K.S. (2018). Staging and grading of periodontitis: Framework and proposal of a new classification and case definition. J. Periodontol..

[29] Tawil G., Barbeck M., Unger R., Tawil P., Witte F. (2018). Sinus Floor Elevation Using the Lateral Approach and Window Repositioning and a Xenogeneic Bone Substitute as a Grafting Material: A Histologic, Histomorphometric, and Radiographic Analysis. Int. J. Oral Maxillofac. Implant..

[30] Trajkovski B., Jaunich M., Müller W.-D., Beuer F., Zafiropoulos G.-G., Houshmand A. (2018). Hydrophilicity, Viscoelastic, and Physicochemical Properties Variations in Dental Bone Grafting Substitutes. Materials.

[31] Barbeck M., Udeabor S., Lorenz J., Schlee M., Holthaus M.G., Raetscho N., Choukroun J., Sader R., Kirkpatrick C.J., Ghanaati S. (2015). High-Temperature Sintering of Xenogeneic Bone Substitutes Leads to Increased Multinucleated Giant Cell Formation: In Vivo and Preliminary Clinical Results. J. Oral Implantol..

[32] Khojasteh A. (2015). Polymeric vs hydroxyapatite-based scaffolds on dental pulp stem cell proliferation and differentiation. World J. Stem Cells.

[33] Panagiotou D., Özkan Karaca E., Dirikan İpçi Ş., Çakar G., Olgaç V., Yılmaz S. (2015). Comparison of two different xenografts in bilateral sinus augmentation: Radiographic and histologic findings. Quintessence Int..

[34] Soldatos N.K., Stylianou P., Koidou V.P., Angelov N., Yukna R., Romanos G.E. (2017). Limitations and options using resorbable versus nonresorbable membranes for successful guided bone regeneration. Quintessence Int..

[35] Briguglio F., Falcomatà D., Marconcini S., Fiorillo L., Briguglio R., Farronato D. (2019). The use of titanium mesh in guided bone regeneration: A systematic review. Int. J. Dent..

[36] Watzinger F., Luksch J., Millesi W., Schopper C., Neugebauer J., Moser D., Ewers R. (2000). Guided bone regeneration with titanium membranes: A clinical study. Br. J. Oral Maxillofac. Surg..

[37] Lim H.-C., Lee J.-S., Choi S.-H., Jung U.-W. (2015). The effect of overlaying titanium mesh with collagen membrane for ridge preservation. J. Periodontal Implant. Sci..

[38] Konstantinidis I., Kumar T., Kher U., Stanitsas P.D., Hinrichs J.E., Kotsakis G.A. (2015). Clinical results of implant placement in resorbed ridges using simultaneous guided bone regeneration: A multicenter case series. Clin. Oral Investig..

[39] Cucchi A., Vignudelli E., Napolitano A., Marchetti C., Corinaldesi G. (2017). Evaluation of complication rates and vertical bone gain after guided bone regeneration with non-resorbable membranes versus titanium meshes and resorbable membranes. A randomized clinical trial. Clin. Implant. Dent. Relat. Res..

[40] Gu C., Xu L., Shi A., Guo L., Chen H., Qin H. (2022). Titanium Mesh Exposure in Guided Bone Regeneration Procedures: A Systematic Review and Meta-analysis. Int. J. Oral Maxillofac. Implant..

[41] Cucchi A., Vignudelli E., Franceschi D., Randellini E., Lizio G., Fiorino A., Corinaldesi G. (2021). Vertical and horizontal ridge augmentation using customized CAD/CAM titanium mesh with versus without resorbable membranes. A randomized clinical trial. Clin. Oral Implant. Res..

[42] Elgali I., Omar O., Dahlin C., Thomsen P. (2017). Guided bone regeneration: Materials and biological mechanisms revisited. Eur. J. Oral Sci..

[43] Atrens A., Song G.L., Liu M., Shi Z., Cao F., Dargusch M.S. (2015). Review of recent developments in the field of magnesium corrosion. Adv. Eng. Mater..

[44] Witte F., Hort N., Vogt C., Cohen S., Kainer K.U., Willumeit R., Feyerabend F. (2008). Degradable biomaterials based on magnesium corrosion. Curr. Opin. Solid. State Mater. Sci..

